# ε-Poly-l-lysine Affects the Vegetative Growth, Pathogenicity and Expression Regulation of Necrotrophic Pathogen *Sclerotinia sclerotiorum* and *Botrytis cinerea*

**DOI:** 10.3390/jof7100821

**Published:** 2021-09-30

**Authors:** Tao Zhou, He Liu, Yuanmin Huang, Zehao Wang, Yuhang Shan, Yan Yue, Zihao Xia, Yue Liang, Mengnan An, Yuanhua Wu

**Affiliations:** College of Plant Protection, Shenyang Agricultural University, Shenyang 110866, China; 2019220498@stu.syau.edu.cn (T.Z.); 2019200124@stu.syau.edu.cn (H.L.); 2019240320@stu.syau.edu.cn (Y.H.); 2020200136@stu.syau.edu.cn (Z.W.); 2019220469@stu.syau.edu.cn (Y.S.); 2020240487@stu.syau.edu.cn (Y.Y.); zihao8337@syau.edu.cn (Z.X.); yliang@syau.edu.cn (Y.L.); wuyh09@syau.edu.cn (Y.W.)

**Keywords:** ε-PL, necrotrophic fungi, transcriptomic analysis, anti-fungal modes of action

## Abstract

Microbial secondary metabolites produced by *Streptomyces* are applied to control plant diseases. The metabolite, ε-poly-l-lysine (ε-PL), is a non-toxic food preservative, but the potential application of this compound as a microbial fungicide in agriculture is rarely reported. In this study, the effect and mode of action of ε-PL on two necrotrophic pathogenic fungi, *Sclerotinia sclerotiorum* and *Botrytis cinerea*, were investigated. The results showed that ε-PL effectively inhibited the mycelial growth of *S. sclerotiorum* and *B. cinerea* with EC_50_ values of 283 μg/mL and 281 μg/mL, respectively. In addition, ε-PL at the dose of 150 and 300 μg/mL reduced *S. sclerotiorum* sclerotia formation. The results of the RNA-seq and RT-qPCR validation indicated that ε-PL significantly regulated the gene expression of critical differential expressed genes (DEGs) involved in fungal growth, metabolism, pathogenicity, and induced an increase in the expression of the fungal stress responses and the detoxification genes. These results provided new insights for understanding the modes of action of ε-PL on *S. sclerotiorum* and *B. cinerea* and improved the sustainable management of these plant diseases.

## 1. Introduction

Plant diseases caused by fungal pathogens result in significant economic losses in agriculture production [1]. *Sclerotinia sclerotiorum* (Lib.) de Bary is a filamentous ascomycete and an important plant pathogen [2,3]. This fungal pathogen poses a threat to dicotyledonous crops such as sunflower, soybean, peanut, oilseed rape, lentils and various vegetables, but also monocotyledonous species such as onion, tulip, and garlic [4]. This fungus can produce sclerotia, which serve as long-term survival structures under adverse environments and play critical roles in disease progression [5,6]. Another necrotrophic pathogen, *Botrytis cinerea*, causes grey mold disease in a variety of fruit and vegetables [7]. It is widely accepted that *S. sclerotiorum* and *B. cinerea* are closely related based on their genome sequences [7,8]. Plant-pathogenic fungi can facilitate an infection in their hosts by the secretion of a wide array of cell-wall-degrading enzymes (CWDEs), including cellulases (glucanase), pectinases (polygalacturonase), glycosidases, xylanases and cutinases [5,6]. In addition, *S. sclerotiorum* and *B. cinerea* are known to produce oxalic acid to promote infection [7]. Some lines of chemical or biological fungicides were reported to suppress gray mold disease caused by *B. cinerea,* while reports of the effective agents on *S. sclerotiorum* are very limited [9].

The large-scale and long-term use of chemical synthetic pesticides may enhance the pesticide resistance of pathogens, reduce pesticide sensitivity, negatively affect the ecological environment and pose a threat to human health [10,11]. Compared with traditional chemical pesticides, the microbial source pesticides have various advantages in biodegradability and environmental compatibility [12,13]. *Streptomyces* species are major members of actinomycetes and can produce a large variety of secondary metabolites with potential anti-microbial activities [13,14]. For instance, ε-poly-l-lysine (ε-PL), produced by *S. albulus* or *S. griseofuscus*, is a homopolymer of L-lysine with a polymerization degree of approximately 25–35 and connected by a peptide bond between the α-carboxy group and the ε-amino group [15,16].

The compound ε-PL is applied as a food preservative that exhibits a good anti-bacterial activity and can be degraded into lysine and absorbed as an essential amino acid by the human body without any harmful influence [17]. Additionally, ε-PL is used as an interferon inducer, drug delivery vehicle and gene delivery vector and used in medical research [18]. The anti-bacterial activity of ε-PL was well investigated using *Escherichia coli*, *Staphylococcus aureus*, and *Pseudomonas aeruginosa* [17,19,20], while recent research focused on its effect on other plant pathogens, including viruses and fungi [21,22,23]. Our previous study showed that ε-PL significantly suppressed the infection of the tobacco mosaic virus (TMV) in *Nicotiana glutinosa*, as well as RNA accumulation in tobacco protoplasts and induced host defensive responses [24,25]. In addition, we indicated that ε-PL effectively inhibited vegetative growth and pathogenicity and affected the respective gene expression of *Alternaria alternata* [21]. Recent studies revealed that ε-PL exhibited effective antifungal activity against *Penicillium digitatum* [22]. Furthermore, ε-PL was reported to effectively inhibit the incidence of grey mold rot on various fruits and vegetables caused by *B. cinerea* [23,26]. The high-throughput sequencing techniques, such as Illumina RNA-seq, provided a powerful tool to investigate the transcriptome variations of the pathogenic fungi in response to biological agents [27,28].

In this research, the microbial source agent ε-PL significantly suppressed mycelial growth and regulated the expression of the critical genes involved in fungal growth, pathogenicity, and the stress responses and detoxification of necrotrophic fungi, *S. sclerotiorum* and *B. cinerea*. Such results provided new insights for the mode of action of ε-PL in the management of plant diseases caused by *S. sclerotiorum* and *B. cinerea.*

## 2. Materials and Methods

### 2.1. Preparation of Microbial Agent ε-PL

The compound ε-PL was identified and purified from *Streptomyces microflavus* var. *liaoningensis* with molecular mass in the range of 3454–4352 Da with approximately 25–35 residues [24].

### 2.2. Pathogenic Fungi and Plants

The pathogenic fungi, *S. sclerotiorum* and *B. cinerea* [29], were preserved in College of Plant Protection, Shenyang Agricultural University, China [29,30]. Mycelia cultured on PDA (potato dextrose agar, 20 g agar powder, 20 g D-glucose, 200 g potato) at 25 °C were used in subsequent experiments of mycelial growth and inoculation experiments. Rapeseed plants (*Brassica napus* cultivar Westar) were grown in an artificial climate greenhouse at a constant temperature of 25 °C, 16 h light/8 h dark.

### 2.3. Antifungal Activity of ε-PL In Vitro

The antifungal activity of ε-PL on *S. sclerotiorum*, as well as *B. cinerea*, was tested by measuring mycelial growth in vitro. The PDA medium containing ε-PL solution was adjusted to the final concentrations of 100, 200, 300, 600 and 1200 μg/mL, while the PDA without ε-PL solution served as a control, respectively. The mycelial plugs (5 mm in diameter) were placed in the center of the PDA plates and cultured in a 25 °C incubator. The growth of fungal colony was measured at 3-day post inoculation (dpi) for *S. Sclerotinia* and 4 dpi for *B. cinerea* with three inoculation replicates of each pathogen; the growth assays were independently repeated three times. The inhibition rate of ε-PL on the mycelial growth of *S. sclerotiorum* and *B. cinerea* was calculated by the following formula [31]: antifungal rate (%) = [(control − treated)/control] × 100%. The effective medium concentration (EC_50_) values of the agent on *S. sclerotiorum* and *B. cinerea* were calculated.

Furthermore, the antifungal activity of ε-PL on sclerotial development of *S. sclerotiorum* was evaluated [32]. Sclerotial formation was estimated in different concentrations (50, 100, 150, 200 and 300 μg/mL) of ε-PL treatment after 9 dpi while the PDA without ε-PL solution served as a control. Sclerotia were collected and air-dried at 70 °C for 4 h and then the biomass was weighed. Antifungal activity estimation was independently conducted five times.

### 2.4. Antifungal Activity of ε-PL on Detached Leaves

Mycelial plugs (5 mm) of *S. sclerotiorum* were directly placed on the excised rapeseed leaves that were prewounded by syringe needle for pathogen inoculation. After 12 hpi, the inoculated leaves were sprayed with a series of concentrations of ε-PL at 200, 400, 600 and 1200 μg/mL while the water spray served as a mock. The inoculated leaves were consistently incubated at 22 °C and the lesion was photographed at 24 hpi. The necrotic lesions were quantified by Assess software (APS Press, St. Paul, MN, USA). Each inoculation with each concentration of ε-PL treatment was performed with five leaves and the inoculation assays were independently repeated three times.

### 2.5. cDNA Library Construction and Illumina Sequencing

Total RNA was extracted from the mock or 280 μg/mL (EC_50_ concentration) of ε-PL-treated *S. sclerotiorum*, as well as *B. cinerea*, collected from a Petri dish at 3 dpi using TRIzon Reagent (TIANGEN, Beijing, China) and subsequently used for RNA-seq. The sequencing libraries were constructed using NEBNext UltraTM RNA Library Prep Kit (NEB, Ipswich, MA, USA) following the manufacturer’s instructions. The mRNA was purified from total RNA using poly-T oligo-attached magnetic beads and fragmented using divalent cations at elevated temperatures in NEBNext First Strand Synthesis Reaction Buffer (5X). The cDNA was synthesized and purified with AMPure XP system (Beckman Coulter, Beverly, NJ, USA) to select approximately 240 bp fragments. The size-selected, adaptor-ligated cDNA was treated with USER Enzyme (NEB, Ipswich, MA, USA) before PCR. Then, the purified PCR products were analyzed by an Illumina sequencing platform (Illumina NovaSeq 6000; Biomarker, Beijing, China).

The raw reads generated by Illumina sequencing were submitted to the Sequence Read Archive database at NCBI (SRA, http://www.ncbi.nlm.nih.gov/Traces/sra, accessed on 19 July 2021) with the SRA BioProject accession number PRJNA749671 (ε-PL or mock treated *S. sclerotiorum* samples); PRJNA749479 (ε-PL- or mock-treated *B. cinerea* samples). The clean reads were mapped to the reference genome sequence using Hisat2 tools soft (http://ccb.jhu.edu/software/hisat2/index.shtml, accessed on 18 February 2021). Differential expression analysis of ε-PL-Ss (*S. sclerotinia* treated with ε-PL) versus mock-Ss (*S. sclerotinia* treated with distilled water), and ε-PL-Bc (*B. cinerea* treated with ε-PL) vs. mock-Bc (*B. cinerea* treated with distilled water) were performed using the DEseq (http://www.bioconductor.org/packages/release/bioc/html/DESeq.html, accessed on 21 February 2021). The differential expressed genes (DEGs) were determined by adjusting the false discovery rate < 0.05. To further analyze these DEGs, Gene Ontology (GO) as well as Kyoto Encyclopedia of Genes and Genomes (KEGG) pathway (http://www.genome.jp/kegg/, accessed on 25 February 2021) were conducted.

### 2.6. Reverse-Transcription Quantitative PCR (RT-qPCR) Assay

The ε-PL (EC_50_ concentration) or distilled water treated *S. sclerotiorum* or *B. cinerea* mycelium were collected at 3 dpi. The total extracted RNA was subjected to reverse-transcription using a FastKing RT Kit (TIANGEN). The primer sets were designed within the CDS region of nucleotide sequences of *S. sclerotiorum* or *B. cinerea*. Then, RT-qPCR analysis was conducted using ChamQ Universal SYBR qPCR Master Mix (Vazyme, Nanjing, China) on StepOne Plus real-time PCR system (Thermo Fisher Scientific). Relative expression of each gene was assessed by 2^−ΔΔC^_T_ method with the normalization using *actin* of *S. sclerotiorum* (SS1G_08733) or *B. cinerea* (BC1G_13894) as a reference gene by three biological replicates.

### 2.7. Statistical Analysis

All of the data were statistically analyzed by one-way analysis of variance (ANOVA) (*p* < 0.05) and then the means were separated by Tukey’s multiple comparison test (*p* < 0.05) using SAS software (version 9.1; SAS Institute Inc., Cary, NC, USA).

## 3. Results

### 3.1. ε-PL Inhibit Growth and Development of S. sclerotiorum and B. cinerea

**Figure 1:**
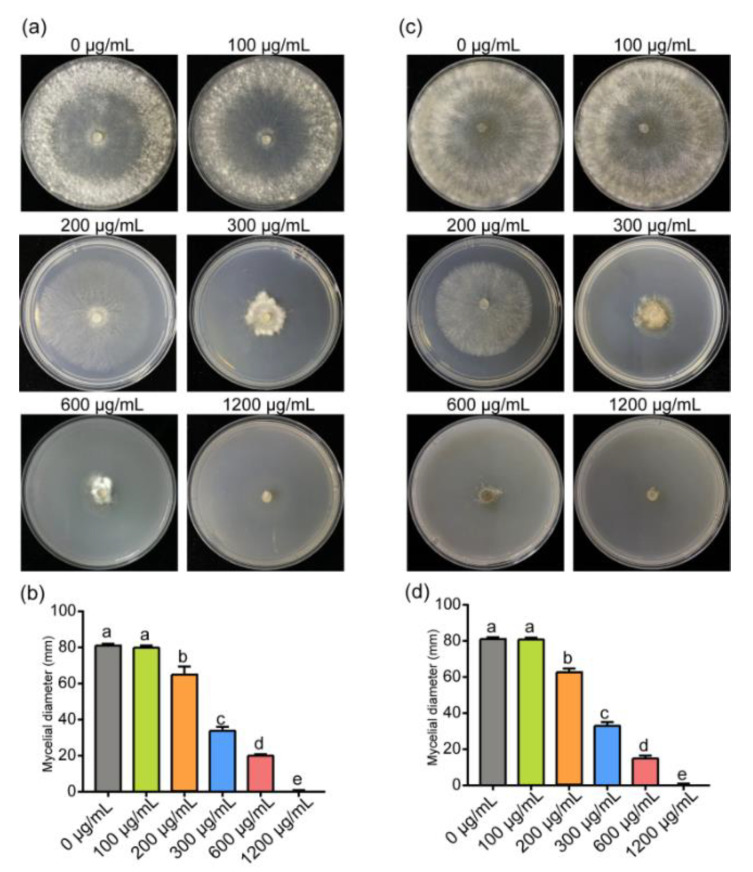
Effects of different concentrations of ε-PL on the mycelial growth of *Sclerotinia sclerotiorum* and *Botrytis cinerea*. (**a**) Colonies of *S. sclerotiorum* treated with ε-PL at concentrations of 0, 100, 200, 300, 600 and 1200 μg/mL at 3 dpi. (**b**) Mycelial growth of *S. sclerotiorum* affected by different concentrations of ε-PL. (**c**) Colonies of *B*. *cinerea* treated with ε-PL at concentrations of 0, 100, 200, 300, 600 and 1200 μg/mL at 4 dpi. (**d**) Mycelial growth of *B. cinerea* affected by different concentrations of ε-PL. Different letters indicate significant differences (*p* < 0.05).

The inhibitory effect on mycelial growth and the sclerotial development of *S. sclerotiorum* at a series of concentrations of ε-PL was investigated. The results indicated that the increased concentration of ε-PL treatment gradually inhibited the growth of *S. sclerotiorum*. Especially, the inhibition of mycelium growth was observed at 100, 200, 300 and 600 μg/mL, while at 1200 μg/mL the mycelium growth was completely inhibited (Figure 1a). Particularly, ε-PL at the concentration of 300 and 600 μg/mL significantly suppressed the mycelial growth of *S. sclerotiorum* (Figure 1b), while 100 μg/mL of ε-PL and lower concentrations did not exhibit an effective inhibitory effect. The inhibition rate of ε-PL at different concentrations on *S. sclerotiorum* is shown in Table S1. For example, the inhibition rates of ε-PL at 200, 300 and 600 μg/mL on *S. sclerotiorum* were 40.35 ± 3.88%, 58.16 ± 1.01% and 75.29 ± 1.22%, respectively. Another necrotrophic fungus, *B. cinerea*, was also used to test the antifungal effect of ε-PL and the inhibition rate caused by the treatment of different concentrations of ε-PL was generally consistent with that of *S. sclerotiorum* (Figure 1c,d). The EC_50_ of ε-PL was determined as 283 μg/mL and 281 μg/mL based on the mycelial growth of *S. sclerotiorum* and *B. cinerea* at the different concentrations of ε-PL treatments, respectively. The results indicated that ε-PL could inhibit the mycelial growth of *S. sclerotiorum* and *B. cinerea*.

**Figure 2:**
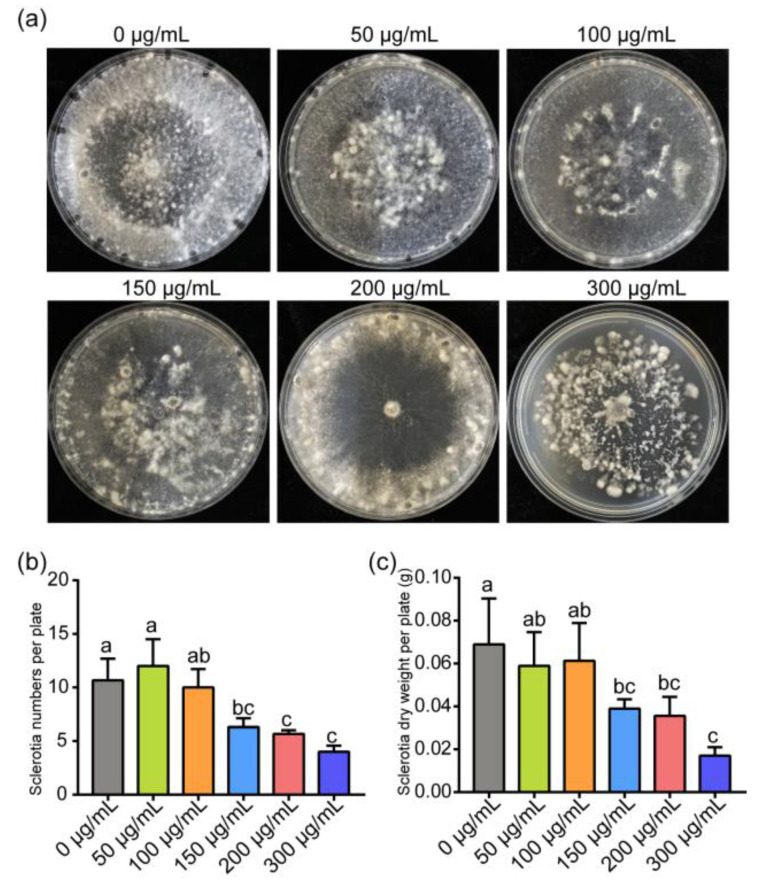
Effects of 0, 50, 100, 150, 200 and 300 μg/mL ε-PL treatment on the number, morphology and dry weight of sclerotia produced by *Sclerotinia sclerotiorum* at 9 dpi. (**a**) ε-PL treatment higher than 150 μg/mL affected the formation and morphology of sclerotia. (**b**) Effect of different concentrations of ε-PL on the number of sclerotia. (**c**) Effect of different concentrations of ε-PL on the dry weight of sclerotia. Different letters indicate significant differences (*p* < 0.05).

Our results also showed that 150 and 300 μg/mL of ε-PL treatment significantly inhibited the sclerotial formation, such as the numbers and the dry weight of sclerotia, but did not affect the sclerotial formation under 100 μg/mL ε-PL treatment (Figure 2).

### 3.2. Inhibition of ε-PL on Infection of S. sclerotiorum and B. cinerea In Vivo

**Figure 3:**
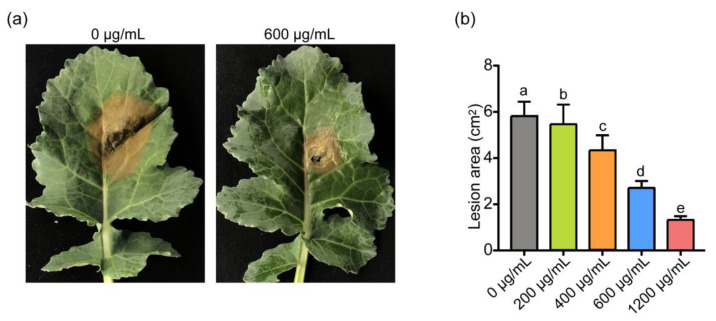
Effects of 0, 200, 400, 600 and 1200 μg/mL of ε-PL on the infection of *Sclerotinia* *sclerotiorum* on rapeseed leaves. (**a**) ε-PL at 600 μg/mL significantly reduced necrotic lesion area caused by infection of *S. sclerotiorum.* (**b**) Effect of different concentrations of ε-PL on the necrotic lesion induced by *S. sclerotiorum.* Different letters indicate significant differences (*p* < 0.05).

Subsequently, the inhibitory effect of ε-PL treatment on the infection of *S. sclerotiorum* was investigated using detached leaves of rapeseed. The results showed that the increased concentrations of ε-PL at 400, 600 and 1200 μg/mL progressively inhibited the necrotic lesion formed by *S. sclerotiorum* (Figure 3). Specially, the 600 and 1200 μg/mL ε-PL concentrations reduced the lesion area by 47.23% and 76.28%, respectively (Figure 3b). Meanwhile, 200 μg/mL and lower concentrations of the agent treatment did not exhibit an observable inhibitory effect (Figure 3b). In addition, the inhibitory effect of ε-PL on *B. cinerea* was tested using tomato and pear fruits. The results indicated the effective inhibitory effect of 600 to 1500 μg/mL of ε-PL on the infection of *B. cinerea* in planta by reducing the area of the necrotic lesion approximately 2- to 5-fold (data not shown), which was generally consistent with previous reports [33].

### 3.3. Transcription Analysis of S. sclerotiorum and B. cinerea Affected by ε-PL

RNA-Seq has recently become a powerful tool for transcriptome profiling in studies monitoring fungal transcriptional responses [27,28]. Based on the inhibition effects of ε-PL on *S. sclerotiorum* and *B. cinerea*, a genome-wide gene expression analysis was conducted to further investigate the effect of ε-PL on the modulation of the critical genes or pathways of *S. sclerotiorum* and *B. cinerea*. The in vitro ε-PL- or mock-treated mycelium of the two pathogenic fungi were collected and subjected to RNA-seq. A total of 24.89 Gb raw reads were obtained and the clean reads were mapped to the reference genome database of *S*. *sclerotiorum* (ASM14694v1) and *B*. *cinerea* (ASM14353v4), respectively. There were 19,508,204 and 22,306,964 clean reads from the ε-PL treatment and control libraries of *S. sclerotiorum*; and 19,850,423 and 20,176,913 clean reads from the ε-PL treatment and control libraries of *B. cinerea,* respectively (Table S2).

**Figure 4:**
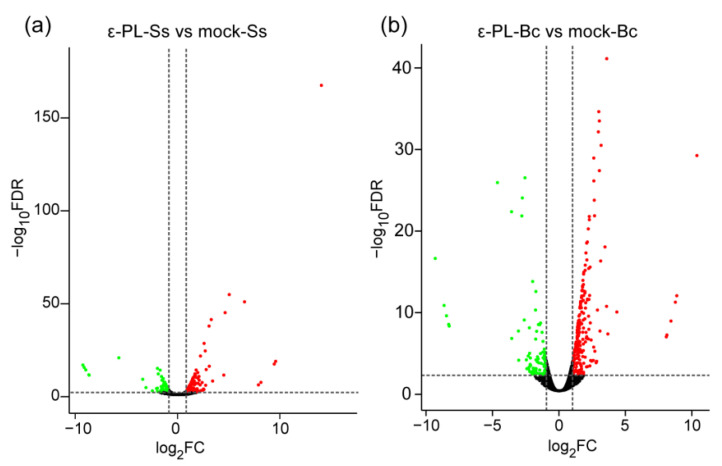
Volcano plots showing differential expressed genes (DEGs) of ε-PL-treated *Sclerotinia* *sclerotiorum* (**a**) ε-PL-Ss vs. mock-Ss or *Botrytis cinerea* (**b**) ε-PL-Bc vs. mock-Bc compared with mock. The red and green colors represent the significantly up- and down-regulated genes, respectively (FDR < 0.05 and |log_2_FC| ≥ 1).

**Figure 5:**
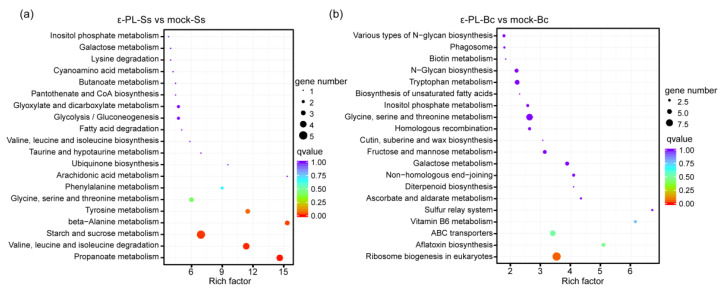
Kyoto Encyclopedia of Genes and Genomes (KEGG) pathways of the enriched DEGs of (**a**) ε-PL-Ss vs. mock-Ss and (**b**) ε-PL-Bc vs. mock-Bc. The rich factor reflects the degree of enriched DEGs in a given pathway. The number of enriched DEGs in the pathway is shown by the circle area, and the circle color represents the ranges of the corrected *p*-value.

Volcano plot, and Log_2_-fold change (Log_2_FC) showed the comparison of DEGs between the mock- and ε-PL-treated group (Figure 4). A total of 262 DEGs were identified in *S. sclerotiorum*, which included 168 up-regulated DEGs and 94 down-regulated DEGs in *S. sclerotiorum* (Table S3). Meanwhile a total of 411 DEGs were identified in *B. cinerea*, including 325 genes which were up-regulated and 86 genes which were down-regulated (Table S4). The DEGs were mainly classified in the terms of biological processes, cellular components and molecular functions in the GO analysis (Figure S1), in which metabolic, cellular and single-organism processes, in the terms of biological process; cell, organelle and membrane components, in the terms of cellular components; and catalytic, binding and transporter activity, in the terms of biological processes, were enriched in *S. sclerotiorum* and *B. cinerea* (Figure S1). Additionally, the total annotation of the DEGs in KEGG pathways were shown (Figure 5). The “ε-PL-Ss vs. mock-Ss”, “starch and sucrose metabolism”, “Valine, leucine and isoleucine degradation,” as well as “propanoate metabolism” were the most enriched pathways. In contrast, the pathways involved in “ribosome biogenesis”, “glycine, serine and threonine metabolism” and “ABC transporters” were enriched in “ε-PL-Bc vs. mock-Bc” (Figure 5).

### 3.4. Gene Expression Involved in the Growth and Pathogenicity, Metabolism, Stress Responses and Detoxification

**Figure 6:**
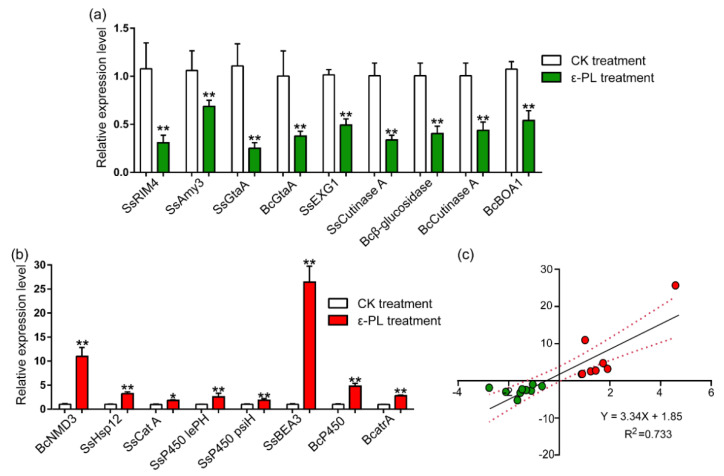
RT-qPCR verification of the regulation of 17 DEGs from ε-PL-treated Sclerotinia sclerotiorum or Botrytis cinerea. (**a**) DEGs mainly involved in fungal growth, or metabolites and pathogenicity, were down-regulated by ε-PL. (**b**) DEGs mainly involved in fungal stress response and detoxification were up-regulated by ε-PL. (**c**) Regression analysis of RNA-seq (independent variable) and RT-qPCR (dependent variable) data to evaluate their correlation by GraphPad Prism 7. Asterisk “*” indicated significant difference (*p* < 0.05) and “**” indicated extremely significant difference (*p* < 0.01) by ε-PL treatment compared with control treatment. Red color represents up-regulated genes while green color represents down-regulated genes.

Metabolism is central to microbial life [34], and the results of RNA-seq showed that a variety of genes involved in carbohydrate or amino acid metabolism were differentially regulated (Figure 6; Table 1, Tables S3 and S4). Several critical DEGs involved in metabolism and fungal growth were listed in Table 1 and the underlined DEGs were subjected to RT-qPCR to verify their expressions. The specific amplification primers were listed in Table S5. The results showed that expression levels of *alpha-amylase A* (*SsAmy3*, SS1G_13472), *meiotic activator RIM4* (*SsRIM4*, SS1G_03997), *glutaminase A* (*SsGtaA*, SS1G_08889 and *BcGtaA*, BC1G_10486) were reduced in *S. sclerotiorum* or *B. cinerea* treated by ε-PL (Figure 6a). Specifically, the expression levels of the *ribosomal export protein NMD3* (BC1G_03554) were increased by ε-PL treatment in *B. cinerea* (Figure 6b). Such results were consistent with the those of the RNA-seq.

The release of toxins or cell-wall-degrading enzymes is the primary infection process of pathogenic fungi [35,36,37]. In this study, ε-PL generally decreased the expression of various genes encoding cell-wall-degrading enzymes, such as cellulase, pectate lyase, cutinase, and polysaccharide monooxygenase (Figure 6; Table 1, Tables S3 and S4). The RT-qPCR analysis confirmed that the expression of several cell-wall-degrading enzyme genes, as well as other genes required for pathogenicity, such as *glucan 1,3-beta-glucosidase* (SsEXG1, Ss1G_09216), *cutinase A* (SS1G_13386; BC1G_02936), *beta-glucosidase I* (BC1G_13346), and *oxidoreductase* (*BcBOA1*, BCIN_01g00010) were significantly decreased by ε-PL in *S. sclerotiorum* or *B. cinerea* (Figure 6a). These results were consistent with the results of the RNA-seq.

The transcriptomic results showed that ε-PL also induced the significant differential expression of genes involved in fungal stress responses, such as heat shock and detoxification proteins, including the cytochrome P450s and ABC transporters in *S. sclerotiorum* and *B. cinerea* (Figure 6; Table 1, Tables S3 and S4). The RT-qPCR were performed to validate the expressions of *heat shock protein 12* (*SsHsp12*, SS1G_05007), *catalase A* (*SscatA*, SS1G_05200), *cytochrome P450 monooxygenases* (*SslepH*, SS1G_00119; *SspsiH*, SS1G_00121; *BcAN1958*, BC1G_u), and *ABC transporters* (*SsBEA3*, SS1G_04757; *BcatrA*, BC1G_02800) in *S. sclerotiorum* or *B. cinerea* treated by ε-PL. The results showed that the expression of these tested genes significantly increased after ε-PL treatment in *S. sclerotiorum* and *B. cinerea* (Figure 6b). Additionally, regression analyses showed that there was a positive correlation between the RNA-seq and RT-qPCR data (Figure 6c).

## 4. Discussion

The ε-PL was mainly applied to inhibit bacteria and was well-characterized as a food preservative [54]. In the previous study, we investigated the functions of ε-PL on the induction of host defense responses against the infection of the tobacco mosaic virus (TMV) [25], as well as the anti-fungal mechanisms of ε-PL on *A. alternata* [21]. The ε-PL was reported to effectively inhibit the incidence of grey mold rot on various fruits and vegetables caused by *B. cinerea* [23,26]. A study of the mode of action indicated that ε-PL treatment could suppress fungal infection by inducing leakages of intercellular electrolytes or proteins and increasing the membrane permeability of *B. cinerea* [23]. Furthermore, ε-PL was also reported to directly act against the pathogenic fungi, *A. alternata*, by disturbing pathogen membrane integrity [55]. In addition, ε-PL showed an inhibitory activity on the spore germination of *Drechslera erythrospila*, *B. cinerea*, and *Phytophthora infestans* [56].

The next-generation sequencing techniques are powerful tools to reveal the transcriptome variations of *S. sclerotiorum* and *B. cinerea* during infection and to respond to fungicides or biological agents [28,35,57]. For example, global gene expression using RNA-seq was performed to reveal the gene regulation of *S. sclerotiorum* during the infection of *Glycine max* [35]. In addition, transcriptome sequencing was used to analyze the gene expression of *S. sclerotiorum* treated with the fermentation broth of *Bacillus amyloliquefaciens* [28]. Transcriptomic analysis was also conducted to analyze critical genes involved in the infection process of *B. cinerea* [58], as well as the resistance-related genes of the *B. cinerea* B05.10 strain in response to fungicide cyprodinil and fenhexamid [57]. In this research, we showed the effective inhibitory effect of ε-PL on *S. sclerotina*, as well as on *B. cinerea*, and compared the effects of ε-PL between two typical necrotrophic pathogenic fungi by revealing regulatory trends on the critical genes and pathways of the pathogen.

Metabolic processes are required for the growth, as well as reproduction, of all kinds of microorganisms [34]. Carbon sources, such as glucose, maltose and fructose, as well as nitrogen sources, could be determining factors affecting fungal growth and sclerotia formation [59]. The results of the RNA-seq showed that many DEGs involved in carbohydrate or amino acid metabolism, such as alpha-amylase, glutaminase and serine carboxypeptidase, were reduced by ε-PL in *S. sclerotiorum* or *B. cinerea*, which indicated that the agent could suppress the basal metabolisms of the fungi, resulting in a decrease in fungal growth and sclerotia formation. Phosphatidylserine decarboxylase (PSDs) can be classified into two types, and the deletion mutants of PSDs cause severe growth defects and the malformation of *Aspergillus nidulans* [39]. Therefore, the inhibitory effect of ε-PL on the expression of *SsPSD* (SS1G_04563) may also result in the decreased fungal growth of *S. sclerotiorum*. The meiotic activator, RIM4, was reported to play an important role in the early events of meiosis in *Saccharomyces cerevisiae* [40]. Here, ε-PL treatment markedly suppressed the expression of the meiotic activator *SsRIM4* (SS1G_03997), which suggested a possible inhibition of fungal cell meiosis by the agent treatment.

Plant cell walls are the first barrier against pathogenic fungal invasion [60]. To overcome the cell wall, *S. sclerotiorum* secretes numerous cell-wall-degrading enzymes (CWDEs) such as polygalacturonases, exo-β-1,3-glucanases, xylanases, and cellulases, which are detected during the early stages of infection [61]. In addition, cutinases are characterized as extracellular serine esterases that break the ester bond of cutin from the cuticle of plant [44]. In this study, the decrease in the gene expression of β-1,3-glucanases, pectate lyase, as well as cutinases (*Sscut* and *Bccut*), by ε- PL can result in a reduction in the pathogenicity of *S. sclerotiorum* and *B. cinerea*. The largest group of proteases (including carboxypeptidases or subtilisin-like proteins) induced during fungal infection are serine proteases, which are characterized as virulence determinants in a large number of plant pathogenic fungi [45]. Here, our results showed that ε-PL suppressed the expression of the serine carboxypeptidase (SS1G_12413), which may reduce the virulence and pathogenicity of the fungus.

Botcinic acid and derivatives produced by *B. cinerea* were characterized as important phytotoxins, inducing host chlorosis or necrosis [62], and the genes involved in botcinic acid biosynthesis were designated as *BcBOA1* to *BcBOA17* [46]. Our results showed that the gene expressions of several *BcBOAs*, such as *BCBOA1* and *BCBOA2*, were markedly decreased by ε-PL, which could effectively suppress the infection of *B. cinerea*. The genes, *SsBOA1* to *SsBOA13*, were identified as having a high similarity with *BcBOA1* to *BcBOA13*, but were probably not related to the biosynthesis of secondary metabolites [46].

Osmotic stress and oxidative stress were often associated with reactive oxygen species (ROS) production [63]. Massive ROS, which led to oxidative bursts, was proven to exhibit a significant antimicrobial activity, such as the inhibition of the spore germination of a number of fungal pathogens [64]. Catalases are ubiquitous enzymes which prevent cell oxidative damage caused by stress responses by degrading hydrogen peroxide with a high efficiency [48]. The heat shock proteins (Hsps) are well-characterized, stress-inducible molecular chaperones, ubiquitously present in all forms of life [65]. Collectively, the up-regulation of genes, such as *Catalase A* and *Hsps* by ε-PL treatment, suggest the significant stress responses induced by the agent.

When exposed to xenobiotics or toxins, fungal cytochrome P450s (CYP450s) play critical roles in phase I of xenobiotic detoxification by converting these compounds to comparatively hydrophilic derivatives [49]. Recent RNA-sequencing and molecular genetics approaches validated that three CYP450s (CYP561, CYP65, and CYP68) were involved in the resistance to multiple fungicide classes mediated by xenobiotic detoxification [49]. Next, the fungus utilizes conjugating enzymes for phase II detoxification and efflux transporters, such as ABC transporters, or major facilitator superfamily (MFS) transporters for phase III detoxification [66]. It was reported that the ABC transporters, AtrA and AtrG, were involved in the azole drug resistance in *Aspergillus oryzae* [53]. In this study, ε-PL induced significant up-regulated expressions in many lines of genes in the cytochrome P450 family, glutathione S-transferase, ABC transporter family and MFS transporters in *S. sclerotiorum* and *B. cinerea*. The results indicated that ε-PL could induce the major detoxification pathways of the fungi.

**Figure 7:**
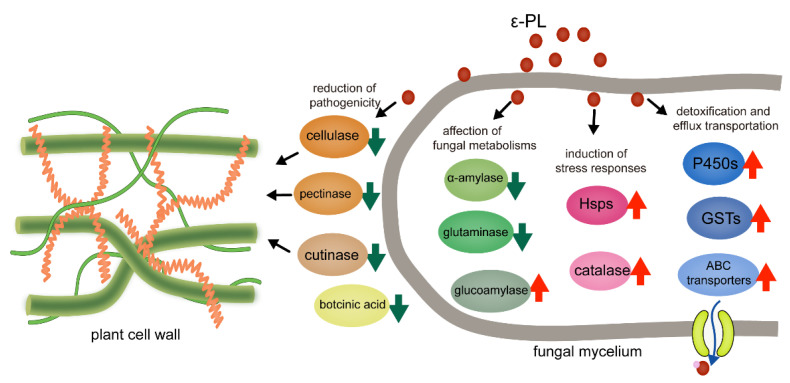
Model for regulation of critical genes involved in fungal metabolisms, pathogenicity, stress responses and detoxification by ε-PL treatment of two necrotrophic fungi.

In this research, we investigated the inhibitory effects and global gene regulation by a microbial source agent, ε-PL, on two representative necrotrophic fungi and collectively summarized our data (Figure 7). The ε-PL was expected to be a green pesticide, which effectively suppressed the mycelial growth and regulated the expression of the critical genes and pathways involved in the pathogenicity, metabolism, stress responses and detoxification of *S. sclerotiorum* and *B. cinerea*. However, the precise inhibitory modes, such as the molecular target of ε-PL on the fungi–host interaction, remain to be further elucidated in future studies. This work will significantly improve the understanding of ε-PL action and the sustainable management of plant diseases caused by *S. sclerotiorum* and *B. cinerea*.

## Figures and Tables

**Table 1:** A general table showing critical DEGs involved in fungus growth and metabolism, pathogenicity, stress response and detoxification induced by ε-PL.

Gene Category	Gene Description	log_2_FC	Regulate	Gene Functions	References
Fungus growth and metabolism					
SS1G_09392	glucoamylase	1.42	up	starch metabolism	[38]
SS1G_04563	phosphatidylserine decarboxylase	−2.51	down	growth and morphogenesis	[39]
SS1G_03997	meiotic activator RIM4	−1.54	down	required for meiosis	[40]
SS1G_13472	alpha-amylase A amy3	−0.68	down	starch metabolism	[41]
SS1G_08889	glutaminase A	−1.65	down	glutamic acid synthesis	[42]
BC1G_10486	glutaminase A	−1.11	down	glutamic acid synthesis	[42]
BC1G_03554	ribosomal export protein NMD3	1.02	up	mRNA and rRNA export	[43]
Pathogenisis					
SS1G_06037	glucan 1,3-beta-glucosidase	−0.75	down	cell wall degradation	[36]
SS1G_09216	glucan 1,3-beta-glucosidase EXG1	−1.05	down	pectin degradation	[36]
SS1G_13386	cutinase A	−2.11	down	cuticle degradation	[44]
SS1G_09821	polysaccharide monooxygenase	1.47	up	lignin or cellulose degradation	[37]
SS1G_12413	serine carboxypeptidase	−1.03	down	virulence determinants	[45]
BC1G_13346	probable beta-glucosidase I	−1.31	down	cell wall degradation	[36]
BC1G_02936	cutinase A	−1.47	down	plant cuticle degradation	[44]
BC1G_09000	probable pectate lyase	−1.08	down	cell wall degradation	[36]
BCIN_01g00010	oxidoreductase BOA1	−2.77	down	putative botcinic acid synthesis	[46]
BC1G_16083	FAD-binding monooxygenase BOA2	−2.81	down	putative botcinic acid synthesis	[46]
Stress response					
SS1G_05007	12 kDa heat shock protein	1.91	up	stress response	[47]
SS1G_05200	catalase A (catA)	0.88	up	stress response	[48]
BC1G_14178	heat shock protein 16	1.35	up	stress response	
BC1G_12146	catalase 7 (cat 7)	1.42	up	stress response	[48]
Detoxification					
SS1G_00119	cytochrome P450 monooxygenase lepH	1.24	up	metabolizing enzymes	[49]
SS1G_00121	cytochrome P450 monooxygenase psiH	0.91	up	metabolizing enzymes	[49]
SS1G_11948	MFS-type transporter SPBC409.08	1.77	up	efflux transport	[50]
SS1G_02254	ABC transporter ARB	0.84	up	efflux transport	[51]
SS1G_04757	ABC transporter BEA3	4.59	up	efflux transport	[51]
SS1G_00727	aldo-keto reductase yakc	3.02	up	detoxification	[52]
BC1G_13302	cytochrome P450 monooxygenase AN1598	1.73	up	metabolizing enzymes	[49]
BC1G_13299	glutathione S-transferase like protein tpcF	1.33	up	conjugating enzymes	[49]
BC1G_00798	MFS-type transporter astH	1.23	up	efflux transport	[50]
BC1G_05984	ABC multidrug transporter atrI	1.81	up	efflux transport	[53]
BC1G_02800	ABC multidrug transporter atrA	1.44	up	efflux transport	[53]

Genes selected for RT-PCR verification were underlined.

## Data Availability

All raw data of RNA-seq are available at Sequence Read Archive (PRJNA749671, PRJNA749479).

## Supplementary Materials

The following are available online at https://www.mdpi.com/article/10.3390/jof7100821/s1, Figure S1: Histogram GO enrichment of DEGs (a) *S**. sclerotiorum* (b) *B**. cinerea*, Table S1: Inhibitory effect of ε-PL against *S**. sclerotiorum* and *B**. cinerea* in vitro, Table S2: Read numbers aligned onto the *S. sclerotiorum* (Ss) and *B. cinerea* (Bc) genome by Illumina sequencing, Table S3: Total DEGs of ε-PL-responsive *S**. sclerotiorum* transcriptome, Table S4: Total DEGs of ε-PL-responsive *B**. cinerea* transcriptome, Table S5: Nucleic acid sequences of oligonucleotide primers used in RT-qPCR analysis.
