# Supplementary material for: ε-Poly-l-lysine Affects the Vegetative Growth, Pathogenicity and Expression Regulation of Necrotrophic Pathogen Sclerotinia sclerotiorum and Botrytis cinerea
Source: J Fungi (Basel). 2021 Sep 30;7(10):821. doi: 10.3390/jof7100821 (PMC8540936; doi:10.3390/jof7100821)
Supplement: Supplementary file 1 [file jof-07-00821-s001.zip › Table S1.pdf]

Table S1. Inhibitory effect of ε - PL against *S. sclerotiorum* and *B. cinerea* in vitro.

**ε-PL concentration (mg/L)**	**Ss inhibitory rate（%）**	**Bc inhibitory rate（%）**
0	0.00±0.00	0.00±0.00
100	18.70±2.80	24.36±2.59
200	40.35±3.88	43.19±5.82
300	58.16±1.01	62.70±3.62
600	75.29±1.22	78.31±1.33
1200	100.00±0.00	100.00±0.00
