# Supplementary material for: ε-Poly-l-lysine Affects the Vegetative Growth, Pathogenicity and Expression Regulation of Necrotrophic Pathogen Sclerotinia sclerotiorum and Botrytis cinerea
Source: J Fungi (Basel). 2021 Sep 30;7(10):821. doi: 10.3390/jof7100821 (PMC8540936; doi:10.3390/jof7100821)
Supplement: Supplementary file 1 [file jof-07-00821-s001.zip › Table S2.pdf]

Table S2. Read numbers aligned onto the *S. sclerotiorum* (Ss) and *B. cinerea* (Bc) genome by Illumina sequencing

Samples	Clean reads	Clean bases	GC Content	%≧Q30
SS ε-PL	19508204	5813095710	46.21%	93.62%
SS mock	22306964	6660780286	46.13%	94.17%
BC ε-PL	19850423	5943072612	46.93%	93.73%
BC mock	20176913	6074441676	46.99%	93.47%
