# Supplementary material for: ε-Poly-l-lysine Affects the Vegetative Growth, Pathogenicity and Expression Regulation of Necrotrophic Pathogen Sclerotinia sclerotiorum and Botrytis cinerea
Source: J Fungi (Basel). 2021 Sep 30;7(10):821. doi: 10.3390/jof7100821 (PMC8540936; doi:10.3390/jof7100821)
Supplement: Supplementary file 1 [file jof-07-00821-s001.zip › Table S5.pdf]

Table S5. Nucleic acid sequences of oligonucleotide primers used to RT-qPCR analysis

Primer name	Sequence of primer (5′-3′)	Amplification sise
SsActin+	AACTGGGATGATATGGAGAAG	114 bp
SsActin-	GTTGGACTTTGGGTTGATTG	
SsCatA+	GACGCCGAAAAACACAAACAC	215 bp
SsCatA-	CTAACCCAACTCGTTGGCGC	
SsHsp12+	GAGAACATCTCTGGAGGAGC	192 bp
SsHsp12-	GTCTTTGTTGCCTGTGAGGG	
SsPSD2+	GACTGGCTCGTACGTTATGC	219 bp
SsPSD2-	GAAAGTTATGATGTGATCAT	
SsEXG1+	GTATTCAAAATGAGCGAGGC	234 bp
SsEXG1-	GTCCCAAAGCTGGGAAATTG	
SsBEA3+	AAGCCTAGATTCCGAAAGCG	224 bp
SsBEA3-	GTCCTATGAGAAAGCATGAA	
SslepH+	CTACAGACGGAAGCCTCACC	205 bp
SslepH-	TCGCTGTATCTATAAGAGCC	
SspsiH +	GGCCTACGATATCCTTCCTG	214 bp
SspsiH -	TCAGATATCGTACGAAGCAC	
Ssamylase+	GGAAGGTGAAGGAGCCGATC	236 bp
Ssamylase-	CAGCGATAGCATTGCCTGTC	
SsRIM4+	TGGTGGTAGCAGAGGCAGAC	210 bp
SsRIM4-	CCTTGACCATCAGTGAAGAC	
SsGluA+	ATGCCTCTTGAAGAATGTGG	236 bp
SsGluA-	CGCGACATGGCTTCAATAGC	
SsCut+	TCCTAACTATTCTCTCTGCC	215 bp
SsCut-	GTAAGTATCGCTGATTCCAG	
BcActin+	CCTAGAGAGATTCCAAGCAG	217 bp
BcActin-	CATGGTTGCAGAGCTTGGCG	
BcatrA+	TGATAATGTGTCCGGATGGG	181 bp
BcatrA-	GTATCCGGTCTTTCGTTGAA	
BcNMD3+	GGCACAAGAACCAAAAACTG	200 bp
BcNMD3-	GGATCCAGGTTTGAGGCGGG	
BcNor19+	ATCAGTAAAGCGGCGGTGAA	198 bp
BcNor19-	CTTCACCCCATCAAACAACC	
BcBOA1+	TGCCCAACTTACAAAGGGAG	202 bp
BcBOA1-	GTGATTCCGTTCTACTCAAA	
BcAN1598+	CGCAAGTCCAACTCTTGCTC	238 bp
BcAN1598-	GGAAACCAGTCGAAGTATCG	
BcGlu+	ATCGCTCGTCCAGTCGTTGG	213 bp
BcGlu-	TAATCTCTAACCGCCGGATC	
BcCut+	GGTGTCTCAGCCGATAGAAC	223 bp
BcCut-	AGGGCTGCCCATGTCGTATC	
Bcβ-glu+	GGAAGGTGAAGGAGCCGATC	200 bp
Bcβ-glu-	CAGCGATAGCATTGCCTGTC	
